# New Jersey State Dental Society

**Published:** 1903-11

**Authors:** 


### NEW JERSEY STATE DENTAL SOCIETY.

The thirty-third annual meeting of the New Jersey State Dental Society convened at the Beach Auditorium, Wednesday morning, July 15, 1903, Dr. Frank L. Hindle, of New Brunswick, the President, in the chair.

After the usual opening preliminaries the President read his address, which was short, and confined to matters of local interest only. This concluded, the meeting adjourned until evening.

The first paper at the evening session was by Dr. Otto E. Inglis, of Philadelphia, upon immediate root-filling. While the paper gave a fair *résumé* of the reasons, *pro* and *con,* for immediate root-filling, and of the conditions when it should be done, and of those requiring caution, it presented nothing especially new. The discussion developed nothing more than has been frequently recorded.

Dr. E. C. Kirk, of Philadelphia, followed with a well-considered paper upon "State Reciprocity of Dental License." He first reviewed the early history of the various settlements which eventually formed the United States, and stated clearly the causes which led these scattered communities to so tenaciously hold and defend the right of self-government. Even during the strenuous struggle of the Revolution this was not lost sight of, and immediately after that fight was won, when it became evident that a closer union was imperative, this right was grudgingly relinquished only so far as the general safety demanded. This was evidenced by the long discussions which preceded the adoption of the Federal Constitution, and the time which elapsed before some States accepted its provisions. Time has not changed this. All legislation which to any degree tends to limit inherent State rights, either by the general government or by the several States, is vigorously contested. Reciprocity of dental State license comes under this head. It cannot be demanded as a right; if it comes at all it must be as an act of courtesy. He suggested that this might be accomplished by a grouping of the States whose requirements were nearly alike and which had adopted much the same standard. Indeed, to some extent this has already been done, and some States have authorized their State boards to recognize the certificates of sister States which in their judgment maintained a standard as satisfactory as their own. By extending this courtesy much just cause of complaint would cease to exist, and as the several groups would eventually find that as their standards were high their certificates of qualification were more valuable and were more widely recognized, this would be to those whose standard was low an incentive to reach a higher level. To accomplish this would require but little new legislation, and would not trench in the slightest degree upon the inherent right of each State to fix its own requirements.

This paper caused an animated discussion, and, while diverse views were presented, the essayist's suggestions were favorably received. The injustice of the present condition of affairs was generally conceded, and it was urged upon all present to use their influence to bring this matter before the National Associations about to assemble, in the hope that some means may be found to bring about a better state of things, and to mitigate the annoyance and constraint a dentist constantly meets when removing from one State to another.

Thursday morning, July 16, Dr. A. W. Harlan, of Chicago, read a paper entitled "The Drug Aspect of Lesions of the Gums." Dr. Harlan stated that, while much attention had been paid to the care of the teeth, the care of the gums had been comparatively neglected. While he would not disparage the importance of keeping the teeth clean and free from deposits of all kinds, he desired to enforce the equal importance of taking care of the gums. Serious results very frequently had their origin in slight injuries to this important tissue. Carelessness in the use of toothpicks, floss silk, toothbrushes, etc., in the hands of our patients, and carelessness in the use of ligatures, wedges, clamps, scaling instruments, etc., in the hands of dentists, is undoubtedly responsible for much injury resulting ultimately in tooth loss. He deprecated the use of so-called breakfast-foods and other predigested, prepared foods, because they did not require mastication. The gums need friction and the teeth exercise, and such foods only should be used as require healthy mastication. The well-being and integrity of the gums is essential to the well-being and integrity of the teeth, and both are essential to good health. The gums protect the tissues upon which the stability of the teeth depends; when this protection is lost, these tissues cease to properly perform their function, they are broken down, the teeth are denuded, loosen, and soon become useless. Lesions of the gums are also due to general lesions, and in turn, in many cases, seriously affect the general health. Mouthwashes are numberless, but, as a rule, not of much use. Friction is essential to the health of the gums and the teeth; nothing can take its place. He especially pleaded for more care to preserve the integrity of the gums and the peridental membrane, and more attention to the first beginning of injuries to these tissues. A touch of the grindstone to relieve malocclusion from a projecting point of a tooth or a filling, a little more care in adjusting appliances, a little more thoughtfulness, when inserting a filling, to avoid, in the effort to reach perfection, undue stress upon the delicate tissues supporting the teeth, and much more attention to see that these tissues are as well taken care of as are the teeth themselves, he urged, would, in many cases, prolong the usefulness of those organs it is the special duty of the dentist to conserve.

In the discussion which followed, the points taken by the essayist met general approval. Incidentally, the advantage of training women as dental nurses was advocated. It was suggested that the lady assistant, by a short course of instruction, would be qualified to give the teeth and gums all needed attention under the supervision of the dentist, and it was also suggested that this division of dental practice should receive legal recognition.

The dentist of to-day is too busy to give the necessary attention to the minor details which are as essential and as important as the most difficult surgical operation. Women versed in the elementary principles of dentistry are to-day most important and necessary adjuncts to the well-regulated dental parlor.

The morning session was concluded by the reading of an essay by Dr. T. D. Shumway, of Plymouth, Mass., upon "Scientific Malletage in filling Teeth." The doctor reviewed the changing views entertained regarding the vitality of the teeth and their relation to the general system since the time Hunter wrote, and argued that, as we now recognize the teeth as vital structures, this should be taken into account in all operations upon them.

The afternoon was devoted to clinics. While the list of clinics upon the programme was a lengthy cne, and promised a very instructive afternoon, comparatively few were on hand. Porcelain inlays took the lead, but nothing especially new was shown. Several cases of regulating were illustrated by plaster casts, which, while interesting as showing what may be accomplished, were instructive only to those who followed closely the operator's explanation. Regarding porcelain work, one point may be of interest. The gentleman demonstrating the Jenkins system contended that low-fusing porcelain should always be fused by heat applied under the matrix; that attempts to fuse it in muffles, such as are provided with furnaces for working high-fusing porcelains, will always be uncertain, if not failures, because the heat is applied all around the matrix, and is not sufficiently under control. When applied at one point, fusion takes place gradually, the porcelain settles down into the matrix, forming a more solid homogeneous mass than when fused in a muffle, and the operator can more readily see when complete fusion has taken place, and thereby avoid overheating. With low-fusing porcelains, he contended, the latitude between just right and too much was limited, very much more so than with the higher-fusing. He suggested that the discrediting of low-fusing porcelains was due to a failure to recognize this, and contended that, used with proper care, low-fusing porcelains were as strong and as reliable as the high, while much more readily worked, and with the same skill more certainly produced accurately fitting inlays.

Dr. William N. Kidder, of Providence, R. I., demonstrated a method of making seamless shell crowns to fit over a model by his hydraulic crown swedger. With this device a model of the tooth over which the crown is to fit is first made of fusible metal. The gold thimble, thoroughly annealed, is fitted over this as well as may be with a smooth-faced hammer, and all surplus gold that safely can be is removed. The model, with the partly fitted crown in place upon it, is now put into a rubber bag about the size of a finger-stall, the open end closed with a clamp, and then placed in the machine. The machine consists of a cylinder into which a screw plug fits. After the crown and model are in place the cylinder is filled with oil, the plug partly screwed in, and a valve opened to allow any air to escape. The valve is then closed and the plug screwed firmly into the cylinder. This produces a very even pressure upon all sides of the contained model, forcing the gold cap into close contact, with no risk of bruising or crushing the fusible metal. It seemed a practical device, and a decided improvement over all others designed for producing a crown to fit over a model.

At the evening session Dr. Genese, of Baltimore, demonstrated a method of mixing plaster with a solution made from rice, by which the plaster is made much harder, and the time of setting more under control than when water is used. With plaster so mixed he quickly made a splint, such as would be used in treating a fractured lower jaw. For this purpose he used a long bag, open nearly its full length on one side, and provided with four tapes to secure it in place. This was filled with plaster mixed with the rice solution and kneaded until about to set. It was then adjusted to place, held securely by the tapes tied over the patient's head, and manipulated so as to make a comfortable fit to the chin. In about five minutes it had set sufficiently hard to be removed, and formed, when fully hard, a rigid, yet easy fitting splint that would, without uneven pressure, firmly support a fractured jaw.

The remaining sessions of the meeting were occupied with society business. On Friday morning the following officers were elected: President, Dr. Herbert S. Sutphen, of Newark; Vice-President, Dr. W. G. Chase, of Princeton; Secretary, Dr. Charles A. Meeker, of Newark; Treasurer, Dr. Henry A. Hull, of New Brunswick.

Sixteen new members were added. The finances were reported to be in a very satisfactory condition, and the Society contemplates in the near future providing for itself suitable head-quarters. The exhibition of dental supplies was a prominent feature of the meeting, and exceeded any previous effort. The large auditorium was fully occupied, and the exhibition, in extent, variety, and interest, fully equalled that of the dental manufacturers recently held in Philadelphia.

